# Factors Associated With HIV Preventive Behavior Among Adults in Majang Zone of Gambella Region, Southwest Ethiopia

**DOI:** 10.3389/fmed.2022.807730

**Published:** 2022-04-19

**Authors:** Tewodros Yosef, Wondimagegn Wondimu, Tadesse Nigussie, Adane Asefa, Qaro Qanche, Besufekad Mekonnen, Gebremeskel Mesafint, Nigusie Shifera, Hailemariam Amsalu, Bayu Begashaw Bekele

**Affiliations:** ^1^Department of Epidemiology and Biostatistics, School of Public Health, College of Medicine and Health Sciences, Mizan-Tepi University, Mizan-Aman, Ethiopia; ^2^Department of Nutrition and Reproductive Health, School of Public Health, College of Medicine and Health Sciences, Mizan-Tepi University, Mizan-Aman, Ethiopia; ^3^Department of Public Health, School of Public Health, College of Medicine and Health Sciences, Mizan-Tepi University, Mizan-Aman, Ethiopia; ^4^Department of Nursing, College of Medicine and Health Sciences, Mizan-Tepi University, Mizan-Aman, Ethiopia; ^5^Department of Biomedical Science, College of Medicine and Health Sciences, Mizan-Tepi University, Mizan-Aman, Ethiopia; ^6^Doctoral School of Health Sciences, University of Debrecen, Debrecen, Hungary

**Keywords:** HIV/AIDS, preventive behavior, Majang zone, Gambella region, Ethiopia

## Abstract

**Background:**

HIV-positive people can spread the virus through unprotected sex; however, HIV can be avoided if populations are educated about the risks. In underdeveloped nations, evidence suggests that the ABC method for HIV prevention is quite effective. As a result, the goal of this study was to determine the magnitude of HIV prevention behavior among adults in the Majang zone of Southwest Ethiopia, as well as the factors that influence it.

**Methods:**

A community-based cross-sectional study was carried out from March 1st to May 31st, 2019. The data were collected through a face-to-face interview using a modified validated questionnaire among systematically selected study participants. The collected data were coded and entered using Epidata manager version 4.0.2.101 and analyzed using SPSS version 21. A logistic regression analysis was computed to determine the association using crude and adjusted odds ratios at 95% confidence intervals. The level of significance was declared at a *p*-value less than 0.05.

**Results:**

Of the 772 adults interviewed, the proportion of adults who had good HIV preventive behavior was 51.8%, 95% CI [48.3–55.3%]. Two hundred forty (31.9%) were used abstinence as a type of HIV preventive behavior followed by being faithful (16.1%) and consistent condom use (7.3%). The study also found that respondents with the age group ≥27 years old (AOR = 1.56, 95% CI [1.3–3.12]), marital status (being married (AOR = 6.30, 95% CI [4.48–11.4]), and divorced/widowed (AOR = 5.50, 95% CI [2.60–12.4]) and having good knowledge of HIV prevention methods (AOR = 2.18, 95% CI [1.71–4.00]) were the factors associated with good HIV preventive behavior.

**Conclusion and Recommendation:**

In the study area, overall HIV prevention behavior was average. The characteristics linked with successful HIV prevention behavior among adults in the Majang community included increasing age, being married or divorced/widowed, and having a solid understanding of HIV prevention approaches. As a result, policy-level and multi-sectorial intervention approaches from all stakeholders are necessary to develop short- and long-term strategies to address the problem and improve the community’s quality of life.

## Introduction

Infection with the human immunodeficiency virus (HIV) is a major global public health issue that affects millions of people each year (1, 2). It is Africa’s second-biggest cause of morbidity and mortality, the fourth major cause of death overall, and the fifth leading cause of death from infectious diseases worldwide (3). The vast majority of HIV-positive people live in low- and middle-income nations (4).

The HIV epidemic, often known as AIDS, varies widely from nation to country and region to region (4), but it is particularly severe in Sub-Saharan Africa, especially Ethiopia, which accounts for more than two-thirds of the world’s HIV load (2, 5, 6). In Ethiopia, overall HIV prevalence has been fluctuating, with most regions indicating decreased trends. In 2005, Gambella, Addis Ababa, and Harari had the highest prevalence rates of 6.0 percent, 4.7 percent, and 3.5 percent, respectively. The highest frequency was observed in the regional state of Gambella, followed by Addis Ababa with 4.8 percent and 3.4 percent, respectively, in the 2016 survey (7).

HIV-positive people can spread the illness through unprotected sex (8), but the disease can be avoided if populations are educated about the risks (9). Abstain, Be Faithful, or Reduce the Number of Sexual Partners, and/or Use a Condom is an HIV/AIDS prevention strategy that is most narrowly defined as Abstain, Be Faithful, or Reduce the Number of Sexual Partners, and/or Use a Condom (10). Abstinence from sexual activity is the most efficient method of preventing pregnancy and sexually transmitted illnesses, such as HIV/AIDS (11). Peer pressure, sex myths, and misconceptions, the influence of drugs and alcohol, and the influence of media are all barriers to sexual abstinence that have become very difficult to overcome (12).

Abstinence-plus interventions emphasize the use of condoms and other safer sex practices in addition to sexual abstinence as the best approach to avoid HIV (13, 14). Being faithful to one’s sexual partner (s) is closely linked to a lower risk of contracting HIV. Couples who are not mutually faithful are more likely to contract HIV than couples who have been together for a long period (15). Condoms can help prevent HIV infection (16–18). Condoms have the greatest preventive impact when they are used frequently rather than infrequently (19). Condom use by HIV-seropositive couples results in near-zero transmission rates to the HIV-seronegative partner (6, 8, 20). Perceived susceptibility and perceived threat of HIV/AIDS showed a correlation with self-efficacy in condoms and their utilization (21).

Correct condom knowledge, as well as favorable attitudes about condom use, is linked to the likelihood of adolescents using condoms (4). Even though most persons were aware of the facts and agreed that condoms are an effective HIV prevention tool, consistent condom use during last sex was particularly low in East Africa (17). Self-efficacy of condoms and their use has been linked to perceived vulnerability and threat of HIV/AIDS (21). The effectiveness of condoms in preventing HIV transmission may be jeopardized if they are misused. Breakage, slipping, leaking, insufficient use, and other issues during the sexual event may jeopardize the condom’s protective function (22).

HIV counseling and condom distribution, which is integrated with other healthcare services and targeted at persons with lower educational attainment, is an essential technique for preventing new HIV infections (23). In underdeveloped nations, evidence suggests that the ABC method for HIV prevention is quite effective. In Uganda, the reduction in HIV prevalence has been dramatic, and it has been connected to changes in sexual behavior (24). As a result, the goal of this study was to determine the magnitude of HIV prevention behavior among adults in the Majang zone of Southwest Ethiopia, as well as the factors that influence it.

## Materials and Methods

### Study Design, Area, and Period

A community-based cross-sectional study was conducted in the Majang zone of Gambella Regional State. Majang zone is found in Gambella regional state and it is among HIV high prevalent areas in Ethiopia (25). It is found 628 km from Addis Ababa, the capital of Ethiopia in the Southwest direction. It has two woredas (namely Godere, and Mangeshi) and one town administration (Meti). Based on the population projection done by the Central statistical agency (CSA) for 2014–2017, the zone had a total population of 79,041, of whom 40,896 were men (26). The study was conducted from March 1st to May 31st, 2019.

### Populations

All adults in the Majang zone were used as the source population. The study population consisted of all randomly selected adults in the Majang zone. Adults between the ages of 18 and 65 were included in the study, while adults who met the inclusion criteria but were extremely ill or unable to interact verbally throughout the data collecting period were excluded.

### Sample Size Determination and Sampling Method

A single population proportion formula was used by taking the following assumptions; the good HIV preventive methods to be 47.6% (27), 95% confidence level, and 5% margin of error.


$$n\,i=\frac{(\text{Z}\alpha /2^{2}p\left(1-p\right)}{{\text{d}}^{2}}=n\,i=\frac{{\left(1.96\right)}^{2}\,0.5\,\left(1-0.5\right)}{{\left(0.05\right)}^{2}}=384$$

After adding 10% for the non-response rate and a design effect of 2; the final computed sample size was 845.

First, we chose 30 percent of Kebeles (the smallest administrative unit) from two woredas and 1 town administration in the Majang zone to identify the calculated sample. In Mangeshi, we took 6 out of 18 kebeles, in Godere, 5 out of 14 kebeles, and in Meti, 1 out of 2 kebeles. A systematic random selection technique was used to choose the sampling unit (households) from the specified kebeles using a sampling frame derived from the health post-family folder registry. Then, if there were more than one eligible participant in the home, the household’s eligible individual was chosen by lottery (28).

### Study Variables and Measurements

The dependent variable was HIV preventive behavior. The independent variables were socio-demographic characteristics (age, sex, marital status, occupation, educational status, income, and residence), and lifestyle factors (alcohol drinking and khat chewing).

Good HIV preventive behavior: A participant who used at least one of the abstinence, be faithful or consistent condom use in the last 1 year (27) and avoid sharing of sharp materials in the last 6 months and tested for HIV in the last 3 months, otherwise poor HIV preventive behavior.

Substance use (alcohol drinking/khat chewing): consuming any substance (alcohol or khat) within the last 1 month/30 days (29).

### Data Collection Instrument and Procedures

The information was gathered using a standardized questionnaire that was created following a review of similar research. The questionnaire was written in English, then translated into the local language, and retranslated back into English by an independent translator to ensure consistency. Experts in the field assessed the content validity. Cronbach’s alpha test was used to determine the analysis’ reliability, and the reliability coefficient was found to be substantial (Cronbach’s alpha: 0.77). Outside of the designated kebeles, a pre-test was conducted on 10% of the sample. The data collectors and supervisors received 1-day training on the objectives and data collection techniques. Face-to-face interviews were used to gather information. The data collection method was monitored by three supervisors. Daily checks were made to confirm the completeness and uniformity of the completed questionnaires.

### Data Processing and Analysis

To reduce errors during data input, the obtained data were coded and entered using Epi-data management version 4.0.2.101, and then analyzed using SPSS version 21. Frequency tables were used to display summary statistics for the independent variables. The link was determined using a binary logistic regression with crude and adjusted odds ratios at 95 percent confidence intervals. Candidates for multivariable logistic regression analysis were independent variables with *p*-values less than 0.25 in bivariate regression analysis. In the multivariable logistic regression analysis, a *p*-value of less than 0.05 was considered significant.

### Ethical Consideration

Mizan-Tepi University’s Research and Community Service Directorate provided ethical approval. A letter of cooperation was issued to the relevant government bodies in the study areas. The participants were also made aware that the information they supplied would be kept private and utilized solely for research purposes. The details of the ethical consideration were better explained in a previous publication (28).

## Results

### Socio-Demographic Characteristics

Of 844 respondents recruited, 772 responded to the interview yielding a response rate of 91.5%. Four hundred fifty-one (58.4%) of the respondents were aged less than 27 years. More than two-thirds (67.7%) and more than half (54.8%) of the respondents were male and protestant religious followers, respectively. More than half (59.6%) and about three-fourths (75.5%) of the respondents were Majang in ethnicity and from rural residences, respectively (Table 1).

**TABLE 1:** Socio-demographic characteristics of the respondents at Majang zone in Southwest Ethiopia, 2019.

Variables	Categories	Frequency (*n* = 772)	Percent
Age	<27 years	451	58.4
	≥27 years	321	41.6
Sex	Male	523	67.7
	Female	249	32.3
Marital status	Single	319	41.3
	Married	373	48.3
	Divorced/Widowed	80	10.4
Religion	Protestant	423	54.8
	Orthodox	238	30.8
	Muslim	111	14.4
Ethnicity	Majang	460	59.6
	Amhara	219	28.4
	Shekicho	55	7.1
	Others*	38	4.9
Level of education	No formal education	80	10.4
	Primary	318	41.2
	Secondary	252	32.6
	College and University	122	15.8
Occupational status	Government employee	123	15.9
	Student	281	36.4
	Merchant	149	19.3
	Farmer	219	28.4
Residence	Urban	189	24.5
	Rural	583	75.5

*Oromo, Tigray, and Keffa.

### Behavioral Profiles and HIV Preventive Behavior

More than one-fourth (27.8%) and 176 (22.8%) of the respondents were alcohol drinkers and khat chewers, respectively. Of 772 respondents, 400 (51.8%) respondents had good HIV preventive behavior. Two hundred forty (31.9%) were used abstinence as a type of HIV preventive behavior followed by being faithful (16.1%) and consistent condom use (7.3%). Almost all (95.6%) didn’t share sharp materials in the last 6 months and 56.9% (439) were tested for HIV in the last 3 months (Table 2).

**TABLE 2:** Behavioral profiles and HIV preventive behavior of the respondents at Majang Zone in Southwest Ethiopia, 2019.

Variables	Categories	Frequency	Percent
Alcohol drinking (*n* = 772)	Yes	215	27.8
	No	557	72.2
Alcohol consumption before	Yes	125	16.2
sexual intercourse (*n* = 215)	No	90	11.7
Khat chewing (*n* = 772)	Yes	176	22.8
	No	596	77.2
Abstinence in the last 1 year (*n* = 772)	Yes	240	31.9
	No	532	68.1
Be faithful in the 1 year (*n* = 772)	Yes	124	16.1
	No	648	83.9
Consistent condom use in the	Yes	56	7.3
last 1 year (*n* = 772)	No	716	92.7
Sharing sharp materials in the	Yes	34	4.4
last 6 months (*n* = 772)	No	738	95.6
Tested for HIV in the last	Yes	439	56.9
3 months (*n* = 772)	No	333	43.1
HIV preventive behavior (*n* = 772)	Yes	400	51.8
	No	372	48.2

### Factors Associated With the HIV Preventive Behavior

After adjusting for age, marital status, khat chewing, and knowledge of HIV preventive methods as confounding variables, age group ≥27 years old (AOR = 1.56, 95% CI [1.3–3.12]), marital status (being married (AOR = 6.30, 95% CI [4.48–11.4]), and divorced/widowed (AOR = 5.50, 95% CI [2.60–12.4]) and having good knowledge of HIV prevention methods (AOR = 2.18, 95% CI [1.71–4.00]) were the factors associated with good HIV preventive behavior (Table 3).

**TABLE 3:** Factors associated with HIV preventive behavior in the Majang zone, Southwest Ethiopia, 2019.

Variables	Categories	HIV preventive behavior		COR (95% CI)	AOR (95% CI)	*P*-value
		Yes	No			
Age group	<27 years	229	222	1	1	
	≥27 years	171	150	2.00 (1.37–2.90)**	1.56 (1.32–3.12)	0.042
Sex	Male	254	269	1	1	
	Female	146	103	1.26 (0.86–1.84)*	1.37 (0.89–2.10)	0.156
Marital status	Single	158	161	1	1	
	Married	200	173	7.14 (4.13–9.61)**	6.30 (4.48–11.4)	<0.001
	Divorced/Widowed	42	38	5.58 (2.56–11.8)**	5.50 (2.60–12.4)	<0.001
Alcohol drinking	Yes	108	107	1	1	0.243
	No	292	205	1.39 (0.96–2.03)*	1.38 (0.88–2.15)	0.162
Khat chewing	Yes	96	80	1	1	
	No	304	292	1.46 (0.99–2.17)*	1.25 (0.77–2.01)	0.368
Knowledge of HIV preventive methods	Poor	250	216	1	1	
	Good	150	156	2.62 (1.48–3.21)**	2.18 (1.71–4.00)	<0.001

*P-value < 0.25 and **P-value < 0.05.

## Discussion

HIV can be avoided if people are educated about the disease (9). ABCs is an HIV/AIDS prevention strategy (10). The adoption of an intervention focused on increasing condom usage, cleaner needle use, and a combination of abstinence, faithfulness, and condom use (ABC) significantly reduced HIV prevalence (9). The goal of this study was to determine the magnitude of HIV prevention behavior among adults in the Majang zone in Southwest Ethiopia, as well as the factors that influence it.

The magnitude of HIV preventive behavior was 51.8%, 95% CI [48.3–55.3%]. This finding was in line with 54.1% in Bale Zone, Ethiopia (30). It was higher than 47.6% in Bench-Sheko Zone (27) and 46.4% in West Gojjam Zone (31) studies in Ethiopia. But, it was lower than 61.1% in the Afar region, Ethiopia (32). The use of a condom regularly can help to minimize the spread of HIV and other sexually transmitted illnesses. Condom use is a cost-effective and cost-saving intervention (every €1 spent on condom distribution saves €5.51) (33). The magnitude of consistent condom use was 7.3%, 95% CI [5.5–9.2%]. It was lower than 46% in the Bench-Sheko Zone (27) and 16.8% in Hawassa (34) studies in Ethiopia, 60% in Cameroon (35), 16.7% in Iran (8), and 53% in the United States (18).

Individuals who had one or no sexual partner were had good knowledge of sexually transmitted infections (36), which is very good for applying good HIV preventive behavior. The magnitude of sexual abstinence was 31.9%, 95% CI [27.6–35.2%]. It was higher than 7.9% in the Bench-Sheko Zone, Ethiopia (27). It was lower than 37.1% in Côte d’Ivoire (11). The magnitude of be faithful was 16.1%, 95% CI [13.5–18.7%]. It was lower than 23.5% in the Bench-Sheko Zone, Ethiopia (27), 52% in Cameroon, and 74% in Zimbabwe (15). The magnitude of tested for HIV in the last 3 months was 56.9%, 95% CI [53.4–60.4%]. It was higher than 43.5% in the Bench-Sheko Zone, Ethiopia (27). Multiple factors contributed to the disparity between this and other research. It could be due to the sample size disparity, the operational definition utilized, or the approach as a whole. Furthermore, economical, behavioral, and sociocultural variables may all play a role in the observed variation.

The respondents’ age was statistically linked to their HIV prevention practice. Respondents under the age of 27 were 1.6 times more likely than those above the age of 27 to use HIV prevention strategies. This research matched that of a study conducted in Ethiopia’s Afar region (32). Individuals appear to be more aware of numerous approaches to avoid HIV/AIDS as they get older (27), which leads to better implementation of HIV preventive behavior.

Married and divorced/widowed respondents had 6.3 and 5.5 times higher probabilities of implementing HIV prevention activity than single respondents, respectively. Being married and being divorced/widowed were both substantially linked to HIV prevention behavior. Fontes MB et al. endorsed this study, which found that being married was linked to HIV prevention practices (37). Yosef T (38) revealed that being single or unmarried had a high chance of acquiring sexually transmitted infections due to the application of poor preventive behavior. This could be because married people are more likely to be faithful to their partners, which could lead to the usage of an HIV prevention strategy. Divorced and bereaved people are also more prone to refrain from sex because they feel demoralized as a result of their sexual partner’s separation.

Good knowledge about STIs including HIV/AIDS was associated with a positive attitude toward condom use (39). Knowing about the disease and the techniques for preventing it is beneficial to HIV prevention. Respondents with a good understanding of HIV prevention behavior were 2.2 times more likely to practice HIV prevention behavior than those with poor knowledge of HIV prevention behavior. Individuals who have comprehensive knowledge about HIV were more likely to practice HIV preventive behaviors than an individual lacking comprehensive knowledge about the disease (40, 41). This could be attributed to the respondent’s degree of practice, which is largely determined by their knowledge level. However, this result was incongruent with Nuked et al., there is no link between knowledge and behaviors/practices (35).

## Limitation of the Study

Participants’ preventative habits were assessed by asking them to recall their previous preventive activities. As a result, there was a chance that recalling bias would be incorporated into the data, necessitating caution when interpreting and applying the findings of this study.

## Conclusion

In the study area, overall HIV prevention behavior was average. The characteristics linked with successful HIV prevention behavior among adults in the Majang community included increasing age, being married or divorced/widowed, and having a solid understanding of HIV prevention approaches. As a result, policy-level and multi-sectorial intervention approaches from all stakeholders are necessary to develop short- and long-term strategies to address the problem and improve the community’s quality of life.

## Data Availability Statement

The original contributions presented in the study are included in the article/supplementary material, further inquiries can be directed to the corresponding author.

## Ethics Statement

The studies involving human participants were reviewed and approved by the Research and Community service directorate of Mizan-Tepi University. The patients/participants provided their written informed consent to participate in this study.

## Author Contributions

TY was involved in the conception, design, and acquisition of data, analysis, interpretation of the results, and drafted the manuscript. WW, TN, AA, QQ, BM, GM, NS, HA, and BB were involved in the design, acquisition of data, and analysis of the results. All authors approved it for publication.

## Conflict of Interest

The authors declare that the research was conducted in the absence of any commercial or financial relationships that could be construed as a potential conflict of interest.

## Publisher’s Note

All claims expressed in this article are solely those of the authors and do not necessarily represent those of their affiliated organizations, or those of the publisher, the editors and the reviewers. Any product that may be evaluated in this article, or claim that may be made by its manufacturer, is not guaranteed or endorsed by the publisher.
